# Effect of change saliency and neural entrainment on flicker-induced time dilation

**DOI:** 10.1167/jov.20.6.15

**Published:** 2020-06-23

**Authors:** Luhe Li, Shogo Ito, Yuko Yotsumoto

**Affiliations:** Department of Life Sciences, The University of Tokyo, Tokyo, Japan

**Keywords:** flicker-induced time dilation, change saliency, neural entrainment, steady-state visual evoked potential (SSVEP), beat

## Abstract

When a visual stimulus flickers periodically and rhythmically, the perceived duration tends to exceed its physical duration in the peri-second range. Although flicker-induced time dilation is a robust time illusion, its underlying neural mechanisms remain inconclusive. The neural entrainment account proposes that neural entrainment of the exogenous visual stimulus, marked by steady-state visual evoked potentials (SSVEPs) over the visual cortex, is the cause of time dilation. By contrast, the saliency account argues that the conscious perception of flicker changes is indispensable. In the current study, we examined these two accounts separately. The first two experiments manipulated the level of saliency around the critical fusion threshold (CFF) in a duration discrimination task to probe the effect of change saliency. The amount of dilation correlated with the level of change saliency. The next two experiments investigated whether neural entrainment alone could also induce perceived dilation. To preclude change saliency, we utilized a combination of two high-frequency flickers above the CFF, whereas their beat frequency still theoretically aroused neural entrainment at a low frequency. Results revealed a moderate time dilation induced by combinative high-frequency flickers. Although behavioral results suggested neural entrainment engagement, electroencephalography showed neither larger power nor inter-trial coherence (ITC) at the beat. In summary, change saliency was the most critical factor determining the perception and strength of time dilation, whereas neural entrainment had a moderate influence. These results highlight the influence of higher-level visual processing on time perception.

## Introduction

Time illusions can provide insight into how the brain integrates and processes temporal information (Merchant, Harrington, & Meck, 2013). Humans are reasonably good at perceiving the duration of a stable visual stimulus. However, in this study, we focused on a prominent time illusion known as flicker-induced time dilation. When a visual stimulus flickers periodically and rhythmically, the perceived duration often exceeds its physical duration (Kanai, Paffen, Hogendoorn, & Verstraten, 2006; Treisman & Brogan, 1992). Although this robust illusion may be useful for understanding time perception, its underlying mechanism remains inconclusive.

One primary account proposes that time dilation results from neural entrainment of the exogenous visual stimulus. Neural entrainment is the alignment and synchronization of endogenous oscillations and external stimulus rhythms (Zoefel, ten Oever, & Sack, 2018). The neural entrainment account is based on the relationship between endogenous neural oscillations and time perception, which was introduced by the striatal beat frequency (SBF) model. Endogenous neural oscillations reflect rhythmic fluctuations in neuronal excitability (Bishop, 1933). Neuron oscillation is naturally tuned to a specific frequency range because of membrane conductance and potential (Buzsáki & Draguhn, 2004; Schroeder & Lakatos, 2009). Because neural oscillations also exist in the absence of sensory inputs, they are referred to as spontaneous and ongoing neural oscillations (Zoefel et al., 2018). The SBF model further related spontaneous neural oscillations to interval timing. In this model, cortical oscillators selective for different frequencies can encode durations, and coincidence detectors read out the pattern of ensemble oscillations (Matell & Meck, 2004; van Rijn, Gu, & Meck, 2014a). Moreover, cortical oscillation phases reset with duration onset and become asynchronous over time because of their different frequencies. They form distinct profiles that can be decoded by striatal medium spiny neurons serving as detectors, which are also specific to particular durations.

Neural entrainment was further implemented in the SBF model to account for circumstances when sensory inputs are present (Hashimoto & Yotsumoto, 2015). The neural entrainment account hypothesized that neural entrainment at a stimulated frequency could change the firing pattern of both the oscillator and detector to cause time dilation. Both model simulation and physiological experiments supported this hypothesis (Hashimoto & Yotsumoto, 2015, Hashimoto & Yotsumoto, 2018). Specifically, the SBF model with neural entrainment simulated the process where entrainment changed the intrinsic oscillator's frequency, which modulated the timing at which each oscillator reached its highest firing probability. As a result, the detector neuron had an earlier activation than the physical duration, leading to time overestimation. In the physiological experiment, they recorded electroencephalograms (EEGs) when participants were performing a duration reproduction task on either a 10 Hz flicker or a constant illuminant stimulus. Results revealed that during flicker presentation and the inter-stimulus interval, a larger amplitude of 10 Hz corresponded to longer reproduced duration, suggesting that flicker-induced neural entrainment affected time dilation. In the auditory domain, a magnetoencephalography study reported that a rate stimulation change modulated both rate perception and neural oscillation frequency, which provided a direct link between neural entrainment and time perception (Herrmann, Henry, Grigutsch, & Obleser, 2013). Based on these studies, we can predict that neural entrainment evokes time dilation at the entrained frequency. In contrast to other accounts that emphasize arousal level and temporal cueing, the neural entrainment account does not assume conscious perception of a stimulus. It predicts that as long as there is neural entrainment, whether caused by exogenous stimuli, nonlinear interaction of neural populations, magnetic or electrical stimulation, or the aftereffect following stimulus offset, there should be time dilation.

In the visual domain, flicker as a sensory rhythmic stimulation is an accepted method for stimulating neural entrainment (Notbohm, Kurths, & Herrmann, 2016). Flickers can also reliably induce steady-state visual evoked potentials (SSVEPs) at the stimulated frequency up to 100 Hz (Gulbinaite, Roozendaal, & VanRullen, 2019; Herrmann, 2001; Norcia, Appelbaum, Ales, Cottereau, & Rossion, 2015). It is thus conceivable that when using flickers to stimulate the visual cortex, SSVEPs may be a marker of neural entrainment. However, whether SSVEPs merely reflect the repetition of event-related responses (ERPs) or genuine neural entrainment, has been under debate (Capilla, Pazo-Alvarez, Darriba, Campo, & Gross, 2011; Keitel, Quigley, & Ruhnau, 2014). One possibility is that although SSVEPs involve superimposition of sensory-evoked responses, they may also entail neural entrainment (Zoefel et al., 2018). Indeed, increasing evidence supports the concept that rhythmic stimuli can induce neural entrainment with weak or no ERPs. For example, in a paradigm that avoids ERPs, neural entrainment can be enhanced in response to imaginary rhythms in both auditory and visual domains (Celma-Miralles, de Menezes, & Toro, 2016). In a paradigm that entails both neural activities, Notbohm et al. (2016) disentangled the repetition and entrainment hypotheses. They found that neural responses induced by rhythmic and arrhythmic flickers were accurately captured by the neural entrainment model rather than the repetition hypothesis, corroborating the theory that flickers induce neural entrainment.

In sum, the neural entrainment account explains flicker-induced time dilation by arguing that flickers may entrain spontaneous neural oscillations that encode durations to exogenous frequencies, thereby advancing detector neuron activation and causing time dilation. Accordingly, the theory predicts that dilation is perceived at the stimulation frequency where neural entrainment is particularly strong. The power of flicker-induced SSVEPs can be used to quantify the neural entrainment strength. Additionally, inter-trial coherence (ITC) is often used as a measure of neural entrainment because it quantifies the phase alignment strength (Zoefel, Archer-Boyd, & Davis, 2018). Therefore, we expected to observe larger power and larger ITC values at the stimulation frequencies, corresponding to the extent of perceived dilation.

Another prominent account suggests that change saliency, the subjective and conscious perception of the flicker, is an indispensable factor in time dilation perception (Herbst, Javadi, van der Meer, & Busch, 2013). The saliency account arose from the finding that human observers were most sensitive to flickers between 8 and 15 Hz, and the dilation effect induced by flickers was saturated from 8 Hz (Kanai et al., 2006; Shady, MacLeod, & Fisher, 2004). Accordingly, the saliency account predicts that if the flicker frequency is larger than the critical fusion threshold (CFF) and, thus, is no longer perceived as flickering, this flicker cannot induce time dilation. Note that SSVEP can still exist above the CFF. According to the saliency account in the Herbst et al. (2013) study, such neural activity cannot induce time dilation without conscious perception of stimulus changes. The authors showed that flickers above the CFF did not induce duration dilation even though weak SSVEPs should still occur. The study concluded that subjective saliency, the conscious perception of stimulus changes, caused the flicker-induced time dilation. However, the saliency account and neural entrainment account are not mutually exclusive because saliency and neural entrainment correlate in such a way that a highly salient stimulus also induces a larger amount of neural entrainment.

Current evidence is inconclusive on whether the neural entrainment account or saliency account provides a better explanation of flicker-induced time dilation. Thus, the present study aimed to separate these two accounts and examine them as independently as possible. The neural entrainment and saliency accounts may reflect different stages in interval timing and may have different effects on the perceived dilation strength.

We initially planned the first two experiments focusing on the effect of subjective saliency on the extent of perceived dilation by manipulating frequencies around the individual CFF. The central feature of these two experiments was that the CFF was measured individually, and the visible/invisible flicker frequencies were determined by subtracting/adding 5 Hz to the CFF. The stimulus was composed of 15 light emitting diodes (LEDs), whose frequency can be specified independently. In this way, we manipulated the level of saliency by controlling the number of visible/invisible flickers in each condition. In the first experiment, we compared three conditions with increasing numbers of visible flickers and decreasing numbers of invisible flickers (i.e. increasing level of saliency) in a duration discrimination task. We hypothesized that increasing the saliency level would result in increased perceived dilation based on the saliency account. To test the effect of the neural entrainment on duration, Experiment 2 equated the number of visible flickers to control the saliency strength between two main conditions. We hypothesized that when the saliency level was similar between conditions, the combined condition would dilate more because it had multiple frequencies that might induce neural entrainment and cause dilation. This turned out not to be the case; thus, we conducted more experiments on neural entrainment.

The following three experiments precluded saliency from neural entrainment using a multi-input frequency-tagging technique to test whether neural entrainment alone was sufficient to induce time dilation. Frequency tagging manipulates the temporal intensity of a stimulus and measures the entrained neural response at the same temporal frequency. Previous studies find that when there are two visual stimulations at different frequencies (f1 and f2), SSVEP at their intermodulation (IM) frequencies (mf1 ± nf2) also arises (Norcia et al., 2015; Regan & Regan, 1988). Specifically, the difference component between two fundamental frequencies (|f1 - f2|) is the beat. Here, to preclude the saliency of flicker changes from neural entrainment, this study used a combination of two high-frequency flickers above the CFF, whereas its beat still theoretically aroused SSVEP at a low frequency. Such combinative high-frequency flickers can arouse neural entrainment at a low frequency while being perceived as static. We tested combinative flickers at two pairs of frequencies (71.4 Hz and 83.3 Hz, 55.5 Hz and 62.5 Hz) and measured EEG of the 55.5 Hz and 62.5 Hz pair in Experiment 4. Furthermore, Experiment 5 used a stableness discrimination task to verify the perceptual stableness of combinative high-frequency flickers.

In summary, we hypothesized that if the neural entrainment account holds, SSVEP power/ITC in the frequency spectrum would predict dilation even without perceiving change saliency. If the saliency account holds, change saliency would always predict perceived dilation.

## Experiment 1

Experiment 1 manipulated frequencies around the CFF to test the effect of change saliency on time dilation. Based on the saliency account, we hypothesized that the condition with more salient flickers would induce longer time perception.

### Methods

#### Participants

Fourteen students from the University of Tokyo, including the second author, participated in Experiment 1 (6 men, 8 women; mean age = 19.4 years, SD = 0.94). All participants had a normal or corrected-to-normal vision. We excluded the data of two participants because of the poor fits to the psychometric functions: their slopes of the psychometric function were smaller than two SDs of the group mean. All participants voluntarily participated in the experiment with 1,000 Japanese Yen (JPY) per hour as payment and provided written informed consent before the experiment. The study was approved by the institutional review boards of the University of Tokyo.

#### Stimuli and apparatus

We presented flickers using a custom-built flicker machine comprising 15 LED fibers, a microcontroller, and a keyboard. In contrast to a conventional monitor with a refresh rate of 60 Hz, the flicker machine had a temporal resolution of 1 ms and was thus able to present LED flickers up to 500 Hz. We inserted the LED fibers into holes with 3 mm diameter and 10 mm depth on a blackboard; the LED spatial arrangements could be changed by using new boards. By connecting the microcontroller to a conventional computer, the frequency and timing of each LED could be predetermined and the LED program could be controlled through the terminal. A keyboard connected to the microcontroller was used to launch the LED program.

Each LED frequency was determined by specifying the duration of on- and off-periods in integral milliseconds. For example, assigning the LED to be on for 10 ms and off for 10 ms repeatedly generated a 50 Hz flicker. In the CFF estimation task, we set the consecutive on- and off-periods to 22, 20, 18, 17, 15, 14, 13, 12, 11, and 10 ms, generating flickers of 22.7, 25, 27.8, 29.4, 33.3, 35.7, 38.5, 41.7, 45.5, and 50 Hz, respectively. In the duration discrimination task, the visible flicker frequencies were determined individually by subtracting 5 Hz from each participant's CFF. Similarly, the invisible flicker frequencies were 5 Hz higher than each participant's CFF. We validated each LED frequency with an oscilloscope. The fixation LED was red with 155 cd/m^2^ luminance, and all other LEDs were orange with 31 cd/m^2^ luminance, measured with the ColorCAL MKII Colorimeter. Illuminance was the same across the three conditions. The viewing distance was approximately 57 cm, and the whole stimulus extended to 10.9° of visual angle vertically and 36.4° horizontally.

#### Procedures

Experiment 1 consisted of the CFF estimation task and duration discrimination task, adapted from Herbst et al. (2013). Both tasks used a temporal two-alternative forced-choice design. Behavioral responses were collected using MATLAB with the Psychophysics Toolbox Version 3 (Brainard, 1997). We conducted the experiment in a darkroom. Participants were instructed to place their head on a chin rest to reduce head movement and to fixate on the central fixation point throughout the experiment.

**Figure 1.**
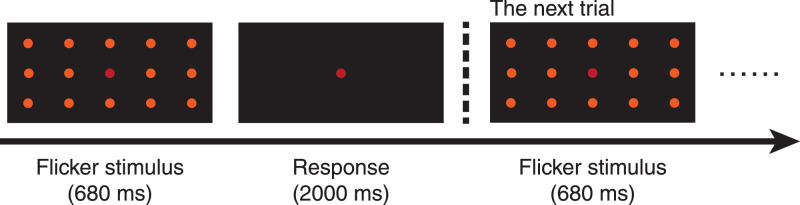
The CFF estimation task in Experiment 1 and Experiment 2.

The CFF estimation task used the method of constant stimuli. As shown in Figure 1, in every trial of the CFF estimation task, the flicker was presented for 680 ms, followed by a 2000 ms response period when only the central red LED was on. Participants needed to press a corresponding key to indicate whether the stimulus was flickering or not flickering. After 2000 ms, the next trial began regardless of whether the participant had responded. There were conditions of flickers at 10 frequencies, with 20 trials in each condition. The order of frequencies was counterbalanced. We grouped a total of 200 trials into four blocks. At the beginning of every block, there was a 2000 ms fixation period for preparation. There was also a tone signaling the end of the block. Participants were permitted a break between blocks.

The CFFs were calculated from the percentage of “stimulus perceived stable” responses at each frequency and we fit these data to a cumulative normal psychometric function using Palamedes toolbox (Prins & Kingdom, 2018). We set the threshold and slope as free parameters and set the guess and lapse rates fixed (guess rate = 0, lapse rate = 0). Based on previous research, the CFF was defined as the frequency at which the flicker was perceived as stable 90% of the time (Herbst et al., 2013). We calculated the visible/invisible flicker frequencies by subtracting/adding 5 Hz from each participant's CFF. To test whether flicker at the CFF-minus-5 Hz was truly flickering to participants, we further examined the CFF estimation task data fitted into the psychometric function. The individual psychometric curve plotted frequencies against the percentage of perceived stable responses. We extracted the percentage of perceived stable responses at the CFF-minus-5Hz frequency to quantify participants’ perception at this chosen frequency. The perceived stable mean percentage was 0.28 (SD = 0.23), far lower than the chance rate of 50%. Results suggested that participants perceived CFF-minus-5 Hz flicker to flash, which substantiated the high saliency of visible flickers used in the following experiments.

**Figure 2.**
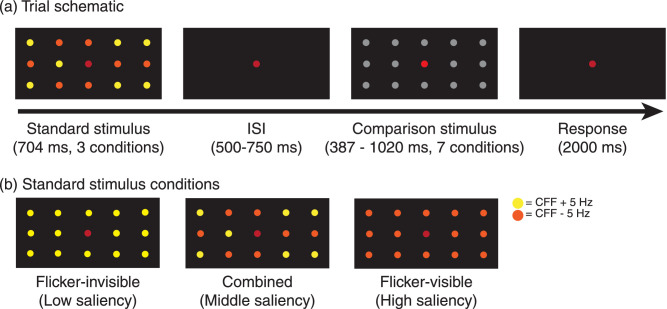
Duration discrimination task and stimulus conditions for Experiment 1. Only the central fixation point was red. Other colors of LEDs are for demonstration purposes; these LEDs were orange in the actual experiment. ISI = inter-stimulus interval; CFF = critical fusion threshold.

In the duration discrimination task (Figure 2a), participants compared the duration of the standard stimuli and comparison stimuli, sequentially separated by a random inter-stimulus interval (500–750 ms). They were instructed to select which one was longer by pressing a corresponding key on a traditional keyboard within a 2000 ms response period, followed by the start of the next trial. If the participant did not respond within the response period, the next trial began automatically when the duration reached 2000 ms.

The standard stimuli were LEDs lit continuously for approximately 704 ms. We presented three frequency conditions (Figure 2b; flicker-invisible, flicker-visible, or combined), featuring different levels of saliency. Note that the actual duration of the standard stimuli had slight variance across participants (*M* = 703.67 ms, SD = 35.30), because the duration was determined by the full frequency cycle in each condition while the visible and invisible flicker frequencies depended on the individual's CFF. In the flicker-invisible condition, apart from the fixation point, 14 other LEDs presented invisible flickers at frequencies slightly above the CFF, resulting in a low level of saliency. Similarly, the flicker-visible condition was composed of 14 visible flickers at frequencies slightly below the CFF, resulting in a high level of saliency. In the combined condition, seven visible and seven invisible flickers were mixed, resulting in a moderate level of saliency. Theoretically, only the combined condition could arouse neural activity at the beat frequency (i.e. 10 Hz) because there were two frequency inputs. As mentioned above, multiple frequency inputs cause IM components by nonlinear processing in the visual system. Such IM components are the indicator of integration perception. For example, previous studies observed IM components when presenting two halves of faces flickering at different frequencies. IM components were only specific to the holistic processing condition when two halves formed the whole face (Boremanse, Norcia, & Rossion, 2013; Boremanse, Norcia, & Rossion, 2014). IM components have also been shown to represent perceptual binding in binocular and interocular rivalry (Sutoyo & Srinivasan, 2009), in perceiving coherent patterns (Cunningham, Baker, & Peirce, 2017), illusory contours (Alp, Kogo, Van Belle, Wagemans, & Rossion, 2016), and symmetry (Alp, Kohler, Kogo, Wagemans, & Norcia, 2018). Therefore, beat as one of IM components is well-established in vision studies.

The comparison stimuli were constantly illuminant LEDs presented for one of the seven durations. We obtained the seven durations of the comparison stimuli by calculating ±45%, ±30%, ±15%, and ±0% of the standard stimulus duration. We counterbalanced the order of the standard stimuli and comparison stimuli across trials. There were 32 trials in each comparison condition; thus, there were 672 trials in total. We randomized the trial order and grouped the trials into 16 blocks.

#### Frequentist statistical analysis

Because the experiment used a within-subjects design with three groups, we used a 1-way repeated-measures ANOVA (rmANOVA) to compare the standardized point of subjective equality (PSE) between groups using JASP (version 0.9.1; JASP Team 2018) software.

#### Bayesian statistical analysis

In addition to a standard frequentist analysis, we conducted a Bayesian statistical analysis to evaluate the null hypothesis (i.e. the finding of no effect) and identify the strength of the evidence, which cannot be achieved by frequentist analysis. For experiments with more than two conditions, we conducted within-group comparisons with corrections for multiple comparisons using JASP. The concern about multiple comparisons centers on the potential inflation of a type I error of a null effect (Neath, Flores, & Cavanaugh, 2018). To deal with this problem, a widely applied correction by Westfall, Johnson, and Utts (1997) calibrated the prior data to regain moderate or high null effects, providing a relatively conservative adjusted posterior probability similar to Bonferroni correction. The resulting BF_10_ represents how strongly the data support the alternative hypothesis that there is a difference between the two conditions. Table 1 shows the Jeffreys (1961) interpretation of the Bayes factor.

**Table 1.** Jeffreys’ Bayes factor interpretation criteria.

BF_10_	Interpretation
10–30	Strong evidence for the experimental hypothesis
3–10	Moderate evidence for the experimental hypothesis
1–3	Anecdotal evidence for the experimental hypothesis
1	No evidence
1/3–1	Anecdotal evidence for the null hypothesis
1/3–1/10	Moderate evidence for the null hypothesis
1/10–1/30	Strong evidence for the null hypothesis

### Results

The average CFF across participants was 36.03 Hz (SD = 2.87). Accordingly, the mean visible flicker frequency was 29.05 Hz (SD = 3.30), and the mean invisible flicker frequency was 40.00 Hz (SD = 3.61).

**Figure 3.**
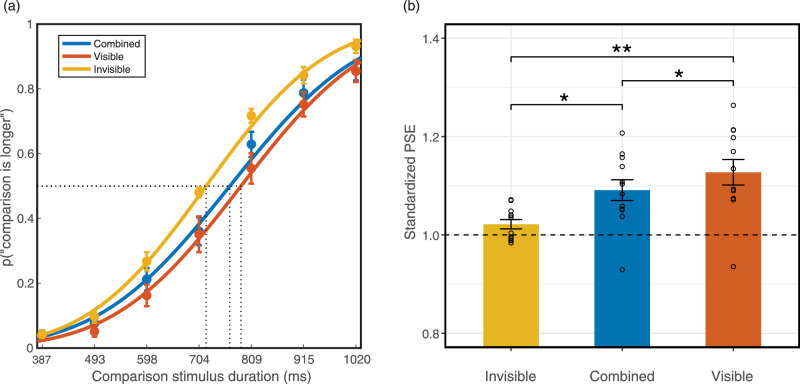
Results of the duration discrimination task in Experiment 1. (a) Averaged psychometric function. The rightward shifts of the curves indicate time dilation. (b) Standardized PSE of each condition. PSE above one indicates time dilation (*n* = 12; * *p* < 0.05; ** *p* < 0.01). Error bars indicate standard errors of the mean.

For each of seven comparison stimulus durations, we calculated the proportion of trials in which the comparison stimulus was judged as longer than the standard stimuli. We applied a cumulative normal psychometric function using Palamedes toolbox to fit the proportion (Prins & Kingdom, 2018), setting the threshold and slope as free parameters and the guess and lapse rate fixed (guess rate = 0, lapse rate = 0). We then calculated the goodness of fit by performing 1000 bootstrap simulations (for the individual parameters and goodness-of-fit, see Supplementary Material Table S1). We fit each condition and each individual and plotted the group-averaged S-shaped psychometric curves (Figure 3). To quantify the extent of dilation, we calculated the PSE for each condition, which was the duration of the comparison stimulus that was perceived to be longer than the standard stimulus at 50% probability of the trials. Therefore, a PSE larger than the standard stimulus duration indicated perceived time dilation and larger PSEs represented greater degrees of time overestimation. To compare the dilation effect across experiments that used a different range of standard stimulus durations, we calculated the standardized PSE by dividing the PSE of each condition by the duration of the standard stimuli. Therefore, a standard PSE larger than one revealed perceived dilation.

**Figure 4.**
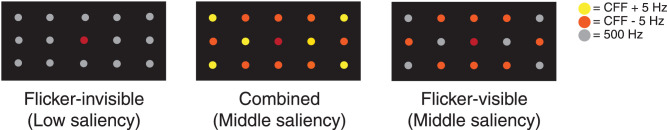
Conditions of the standard stimuli in Experiment 2. Except for fixation, all other colors are demonstrative. Stimuli were orange in the actual experiment. CFF = critical fusion threshold.

In the rmANOVA, the Mauchly's test of sphericity was significant (*p* = 0.04), so the Greenhouse-Geisser correction was used. There was a significant effect of flicker condition on PSE (*F*_(1.35, 14.88)_ = 15.19, *p* < 0.001, *Ƞ^2^_p_* = 0.58). Flicker conditions accounted for 58.0% of the PSE variance. Bayes factors were computed using within-group comparisons with corrections for multiple comparisons. Three two-tailed planned comparisons with Bonferroni corrections revealed that the flicker-visible condition PSE (*M* = 1.13, SD = 0.09) was significantly larger than the combined condition PSE (*M* = 1.09, SD = 0.07, *p* = 0.037, Cohen's d = 0.86, *BF_10_* = 4.96) and flicker-invisible condition PSE (*M* = 1.02, SD = 0.03, *p* = 0.004, Cohen's d = 1.24*, BF_10_* = 33.16). That is, the flicker-visible condition induced a significant perceived time dilation compared with the other two conditions. The combined condition PSE was also significantly larger than the flicker-invisible condition PSE (*p* = 0.014, Cohen's d = 1.02, *BF_10_* = 10.89).

### Discussion

In the duration discrimination task, salient conditions (i.e. the flicker-visible and combined conditions) were perceived to be longer than their veridical duration. Without change saliency, even if the flicker frequencies were only slightly above the CFF, the flicker-invisible condition still failed to cause any dilation. The comparison between the flicker-invisible condition and the other two salient conditions highlighted the importance of change saliency in perceiving flicker-induced time dilation. Moreover, higher levels of saliency induced longer dilation, whereas moderate levels of saliency induced moderate dilation. These results supported the saliency account, which proposes that stimuli with more salient changes induce stronger time dilation. However, we could not draw inferences on the effects of the beat from these results, as there were more visible flickers in the flicker-visible condition than in the combined condition. Therefore, Experiment 2 controlled the number of visible flickers in these two conditions and examined the beat effect.

## Experiment 2

Experiment 2 was adapted from Experiment 1 by controlling the saliency level between the flicker-visible and combined conditions. In Experiment 1, the number of visible flickers differed between the flicker-visible and combined conditions, resulting in increased saliency for the flicker-visible condition. To evaluate the effect of the beat, we equated the number of visible flickers between these two conditions. Because the combined and flicker-visible conditions had similar levels of moderate change saliency, we hypothesized that both conditions would cause time dilation when compared with the flicker-invisible condition. Importantly, because only the combined condition contained the beat from two fundamental frequencies, it was hypothesized that the combined condition would induce longer dilation than the flicker-visible condition based on the neural entrainment account.

### Methods

#### Participants

Twelve students from the University of Tokyo with normal or corrected-to-normal vision participated in Experiment 2, including the second author (all men; mean age = 21.0 years, SD = 1.86). Three of these students also participated in Experiment 1. All participants voluntarily participated in the experiment with 1000 JPY per hour as payment and gave written informed consent before the experiment. The study was approved by the institutional review boards of the University of Tokyo.

#### Stimuli, apparatus, and procedures

The task was the same as that in Experiment 1, except for the standard stimuli conditions (Figure 4). The stimuli in the flicker-visible condition were eight visible flickers and six stable LEDs. We set the stable LEDs to 500 Hz instead of 0 Hz to maintain the same duration and intensity of light across conditions. For example, the 500 Hz flicker was composed of interchanging 1 ms lights on, and 1 ms lights off, so that the cumulative duration of the light being on was one half of the entire stimulus duration. Conversely, 0 Hz meant the light was on for the entire duration, which provided longer and stronger stimulation than did flickers. Similarly, the combined flicker condition included eight visible flickers and six invisible flickers. In the flicker-invisible condition, 14 stable LEDs replaced all invisible flickers as the control.

### Results

The average CFF across participants was 32.36 Hz (SD = 1.92). Accordingly, the visible flicker mean frequency was 28.44 Hz (SD = 1.29), and the invisible flicker mean frequency was 37.91 Hz (SD = 1.23). Following the same method used in Experiment 1, we used the individual psychometric function in the CFF estimation task to calculate how often participants perceived flickering at the CFF-minus-5 Hz flicker. The perceived stableness mean percentage was 0.17 (SD = 0.16, *n* = 12), far lower than the chance rate at 50%. This result suggested that participants perceived visible-flickers at CFF-minus-5 Hz, indicating high saliency. Moreover, because participants had different standard stimulus durations based on their CFF, the mean of standard stimulus durations used in the duration discrimination task was 716.7 ms (SD = 33.2).

**Figure 5.**
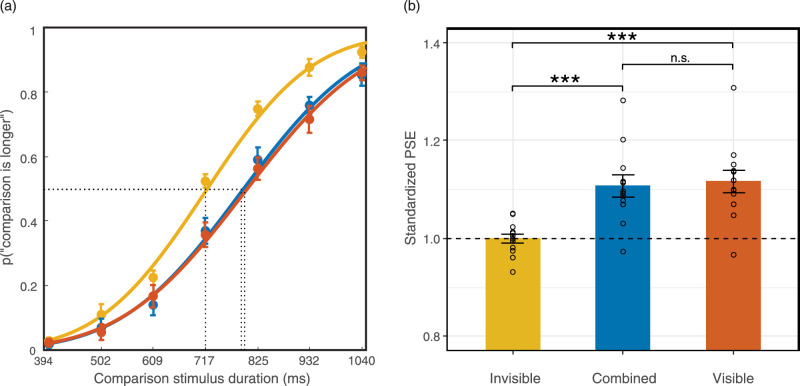
Results of the duration discrimination task in Experiment 2. (a) Averaged psychometric function. The rightward shifts of the curves indicate time dilation. (b) Standardized PSE of each condition. PSE above one indicates time dilation (*n* = 12; *** *p* < 0.001). Error bars indicate standard errors of the mean.

For the duration of the discrimination task, we fit the ratio of perceiving the comparison stimulus as longer into a cumulative normal psychometric function. We set the threshold and slope as free parameters, and the guess and lapse rates fixed (guess rate = 0, lapse rate = 0.02). We fit each condition and each individual (for the individual fit and parameters, see Supplementary Material Table S2) and calculated PSEs from the psychometric curves (Figure 5). In a 1-way rmANOVA, Mauchly's test of sphericity was significant (*p* = 0.01); thus, we applied the Greenhouse-Geisser correction. There was a significant effect of flicker condition on PSE (*F*_(1.309, 14.397)_ = 26.64, *p* < 0.001, *Ƞ^2^_p_* = 0.708). Flicker conditions accounted for 70.8% of the PSE variance. Three two-tailed planned comparisons with Bonferroni corrections revealed that after controlling for the extent of saliency, the flicker-visible condition PSE (*M* = 1.13, SD = 0.08) did not significantly differ from the combined condition PSE (*M* = 1.12, SD = 0.08, *p* = 0.97). BF_10_ for this comparison was 0.45, which provided anecdotal evidence for the null hypothesis. Nevertheless, the PSEs of both conditions were still larger than the flicker-invisible condition PSE (*M* = 1.01, SD = 0.036, both *p* < 0.001, *BF_10_* > 100).

**Figure 6.**
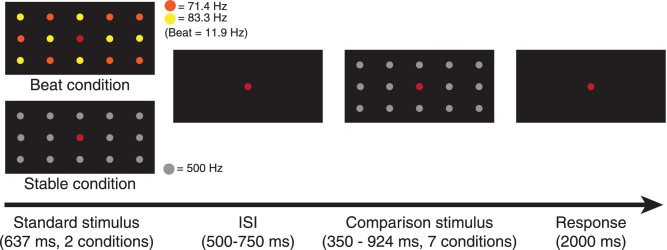
Task schematic and stimulus conditions for Experiment 3. Only the central fixation point was red. Other LED colors are for demonstrative purposes. Stimuli were presented in orange. ISI = inter-stimulus interval.

### Discussion

Contrary to our hypothesis, the extent of dilation in the combined condition was similar to that in the flicker-visible condition. Collectively, the results of Experiments 1 and 2 suggest that the amount of dilation was correlated with the number of visible flickers, implying that perceived saliency is a critical factor for perceiving flicker-induced dilation. However, we did not observe a beat effect that assumed more dilation in the combined condition.

## Experiment 3

Experiment 3 examined the potential effect of neural entrainment alone, precluding saliency. We exploited the beat frequency of the combinative high-frequency flickers to segregate change saliency and neural entrainment. We hypothesized that such beat conditions would induce time overestimation compared with the stable control condition if neural entrainment at the beat frequency alone could increase time perception.

### Methods

#### Participants

Ten students from the University of Tokyo, including the second author, participated in Experiment 3 (7 men, 3 women; mean age = 20.4 years, SD = 1.12). Three of these students also participated in Experiments 1 and 2. All participants voluntarily participated in the experiment with 1000 JPY per hour as payment and gave written informed consent before the experiment. The study was approved by the institutional review boards of the University of Tokyo.

#### Stimuli and procedures

Experiment 3 only used the duration discrimination task from previous experiments because we wanted to focus on frequencies that were high above the CFF (Figure 6). There were two conditions of standard stimuli: beat condition and stable condition, both with durations of 637 ms. The beat condition was a combination of 71.4 Hz and 83.3 Hz flickers, which were both higher than the CFFs that were typically around 30 to 40 Hz in our experimental setting. The beat frequency (difference frequency) was 11.9 Hz, which was substantially lower than the CFF. The stable condition and comparison stimuli all used stable light at 500 Hz. The comparison stimuli had seven durations (±45%, ±30%, ±15%, and ±0% of the standard stimulus duration). Participants compared which stimulus was longer and responded by pressing a key. There were 448 trials and eight blocks in total, counterbalanced across blocks and participants.

### Results

**Figure 7.**
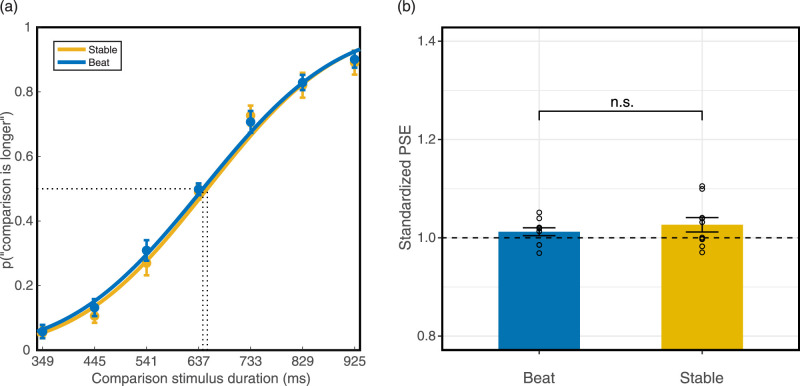
Results of the duration discrimination task in Experiment 3 (*n* = 10). (a) Averaged psychometric function. The absence of the rightward shift of the curve indicates no perceived time dilation. (b) Standardized PSE of the 637 ms standard duration among two conditions. PSE above 1 indicates time dilation. Error bars indicate standard errors of the mean.

All participants reported that the flickers appeared the same so that they could not distinguish conditions. We fit the proportion of trials where the comparison stimulus was perceived to be longer into a cumulative normal psychometric function. We set the threshold and slope as free parameters, and the guess and lapse rates fixed (guess rate = 0, lapse rate = 0). We fit each condition and each individual (for the individual goodness-of-fit and parameters, see Supplementary Material Table S3) and calculated standardized PSEs from the psychometric curves (Figure 7).

**Figure 8.**
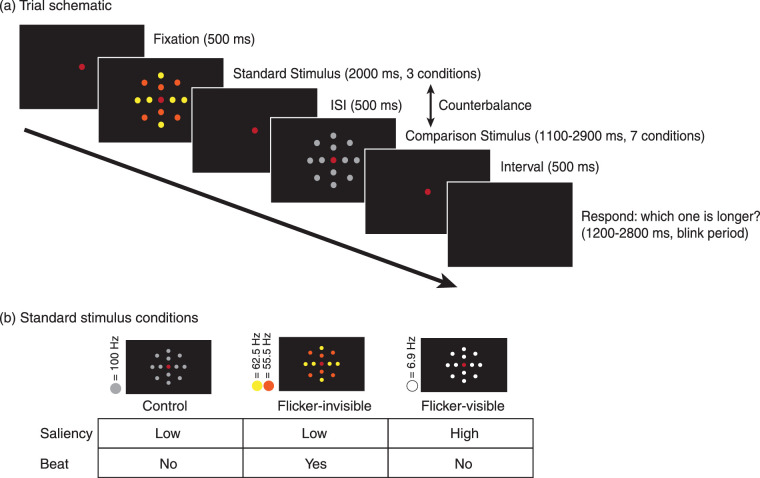
Task schematic and standard stimulus conditions in the duration discrimination task in Experiment 4. Only the central fixation point was red. Other LED colors are for demonstrative purposes. Stimuli were presented in orange.

A one-tailed paired samples *t*-test revealed no significant difference between the beat condition standardized PSE (*M* = 1.01, SD = 0.03) and stable condition PSE (*M* = 1.02, SD = 0.05 *t*_(9)_ = -0.94, *p* = 0.372). A Bayesian paired samples *t*-test indicated that *BF_10_* = 0.44, providing anecdotal evidence for the null hypothesis that the two conditions, were the same.

### Discussion

No duration overestimation was detected in the beat condition, failing to support the neural entrainment account. This finding could result from the weak entrainment of high-frequency flickers. As mentioned above, the neural entrainment strength and SSVEP amplitude decreased as frequency increased (Herrmann, 2001; Pastor, Artieda, Arbizu, Valencia, & Masdeu, 2003). The frequency pair above 70 Hz may be too weak to arouse neural activity at the beat frequency and influence time perception. Further, because integration processing is necessary to generate the beat, perceived combinative high-frequency flickers may not have been sufficiently coherent. Therefore, we conducted Experiment 4 taking these two possibilities into consideration to further investigate the beat effect.

## Experiment 4

Experiment 3 did not support the neural entrainment account. However, it was unclear whether the behavioral findings resulted from weak entrainment or the beat absence. Experiment 4 used EEG measures to examine neural evidence for the beat. We also adjusted parameters to strengthen neural entrainment. Based on the neural entrainment account of time dilation, we hypothesized that the combinative high-frequency flickers would increase perceived durations. We also hypothesized that combinative high-frequency flickers would demonstrate stronger neural entrainment (e.g. larger power or ITC) at the beat frequency and fundamental frequencies.

### Methods

#### Participants

Seventeen participants with normal or corrected-to-normal vision were recruited (8 men and 9 women). All participants were students from the University of Tokyo, including the second author, aged between 23 and 30 years (*M* = 24.41, SD = 1.77). All students voluntarily participated in the experiment with 1500 JPY per hour as payment and gave written informed consent before the experiment. The study was approved by the institutional review boards of the University of Tokyo.

#### Stimuli and apparatus

There were 14 LEDs used in Experiment 4, of which 13 LEDs formed the stimulus, and one was connected to the EEG trigger box. The trigger box was built with a photocell attached to one LED, which sent transitor to transitor logic (TTL) signals upon light detection to the EEG acquisition system. The trigger-LED was set to illuminate for 5 ms at the beginning and end of every stimulus interval, as well as at the trial beginning.

We introduced three major changes in stimuli to induce stronger neural entrainment (Figure 8). First, the flicker pair frequencies in the flicker-invisible condition (referred to as the beat condition in Experiment 3) were 55.5 and 62.5 Hz, because lower frequencies demonstrated higher power in a previous study (Herrmann, 2001). This pair was still above the CFF and utility frequency (50 Hz) that may contaminate EEG data. Second, we added another flicker-visible condition for comparison that set all flickers around the beat frequency of the flicker-invisible condition, which was 6.9 Hz. We set the stable light in the control condition and comparison stimuli to be 100 Hz and 125 Hz, respectively. Therefore, the on- and off-periods of 72, 9, 8, 5, and 4 ms were used to generate flickers of 6.9, 55.5, 62.5, 100, and 125 Hz, respectively. Third, to increase the holistic effect that was critical to inducing the beat, the viewing distance was increased to 100 cm, and the size of the stimulus was decreased; thus, 13 LEDs were rearranged into a compact 0.9° radius circle.

#### Procedures

The task was similar to that in Experiment 3, except for the following adaptations for EEG acquisition. We conducted the experiment in a dark, sound-proof room. Participants were seated in a comfortable chair while keeping their heads on a chin rest to reduce head movements. After completing 10 practice trials, the experimenter applied the 64-sensor HydroCell Sensor Nets (Electrical Geodesics Inc.) to the participant and kept all electrode impedances below 50 kΩ. We recorded EEG data while the participant was performing the duration discrimination task.

At the beginning of every block, a 3-second fixation point was presented as a preparation cue for participants. The first stimulus would appear and remain, followed by a 500 ms inter-stimulus interval, and then the second stimulus. Between the second stimulus and response period, there was a 500 ms gap when only the fixation point was presented to avoid edge contamination in the power analysis. In the following response period that varied from 1200 ms to 2800 ms, participants were asked to select the stimulus duration that was longer by pressing corresponding keys. They were also instructed to only blink during the response period to avoid data contamination.

We decreased the trial number to 672 to shorten the experiment. Trials were randomized and divided into 14 blocks (5 minutes each) and were separated into two sessions on different days to reduce fatigue. Between blocks, participants were allowed to take a break of their desired duration.

#### EEG acquisition and pre-processing

The EEG data were simultaneously recorded by Net Station 5 (Electrical Geodesics Inc.), with 64 electrodes at a sampling rate of 1000 Hz; a 50 Hz notch filter was applied upon export. Data were analyzed offline by EEGLAB (Delorme & Makeig, 2004). We applied a second-order IIR Butterworth bandpass filter between 0.5 and 100 Hz to the data. Data were re-referenced to the average and epoched into intervals between 200 and 2000 ms around the standard stimulus onset. For seven participants, we interpolated one or two noisy channels around the ear or at the frontal area by spherical spline interpolation. None of the interpolated electrodes were in the occipital region. Because interpolations cause rank deficiency and would compromise independent component analysis (ICA), we adjusted the rank by reducing the data dimension using a pca parameter when running ICA. Trials with artifacts, including blinks and eye-movements, were detected and removed if the peak-to-peak threshold was larger than 100 µV both automatically and manually. We applied ICA to the remaining data. By visual inspection, ICA components that showed great power between 20 to 30 Hz and that mainly distributed around the frontal and temporal region were identified as artifacts and removed.

### Results

#### Behavioral results

**Figure 9.**
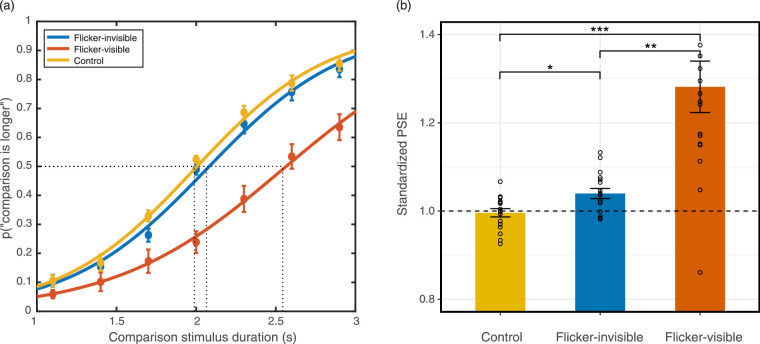
Results of the duration discrimination task in Experiment 4. (a) Averaged psychometric function. The rightward shift of the curve indicates time dilation. (b) Standardized PSE of the 2-second standard duration among three conditions. PSE above 1 indicates time dilation (*n* = 17; *p* < 0.05; **: *p* < 0.01; ***: *p* < 0.001). Error bars indicate standard errors of the mean.

Except in the flicker-visible condition, all participants reported that the stimuli appeared stable, and were unable to distinguish between the stable and the flicker-invisible conditions. We fit the ratio of perceiving the comparison stimulus as longer into a logistic psychometric function, set the threshold and slope as free parameters, and the guess and lapse rates fixed (guess rate = 0, lapse rate = 0.02). We fit each condition and each individual (for individual fit and parameters, see Supplementary Material Table S4) and PSEs were calculated from the psychometric curves (Figure 9).

An rmANOVA was conducted to compare the average standardized PSEs between groups. Mauchly's test of sphericity was significant (*p* < 0.001); thus, we used the Greenhouse-Geisser correction. There was a significant effect of flicker condition on PSE (*F*_(1.067, 17.076)_ = 19.92, *p* < 0.001, *Ƞ^2^_p_* = 0.555). Flicker conditions accounted for 55.5% of the PSE variance. We calculated the Bayes factor using within-group comparisons with corrections for multiple comparisons. Three two-tailed planned comparisons with Bonferroni corrections revealed that the flicker-invisible condition PSE (*M* = 1.04, SD = 0.05) was significantly larger than the control condition PSE (*M* = 1.00, SD = 0.04, *p* = 0.012). Cohen's d for this comparison was 0.81, suggesting a large effect. BF_10_ for this comparison was 11.46, revealing strong evidence supporting the alternative hypothesis that the two groups were different. Consistent with the previous experiments, the flicker-visible condition PSE (*M* = 1.28, SD = 0.24) was significantly larger than both the control condition PSE (*p* = 0.001, Cohen's d *=* 1.14*, BF_10_* = 129.03) and flicker-invisible condition PSE (*p* = 0.002, Cohen's d = 1.03*, BF_10_* = 56.94).

We further conducted a one-tailed one-sample *t*-test, testing the null hypothesis that the flicker-invisible condition PSE comes from a population with a mean equal to 1, against an alternative hypothesis that the mean is > 1. The results rejected the null hypothesis (*t_(__16)_* = 3.46, *p* = 0.002, Cohen's d = 0.84), revealing that the flicker-invisible condition PSE (*M* = 1.04, SD = 0.05) was significantly larger than 1, suggesting perceived dilation.

Furthermore, because the slope of psychometric function represents sensitivity, we conducted an rmANOVA to compare the slope among the three groups. Although there was a significant effect of conditions on slope (*F*_(2, 32)_ = 4.23, *p* = 0.02, *Ƞ^2^_p_* = 0.21), two-tailed planned comparisons with Bonferroni corrections did not find significant difference between the flicker-invisible condition (*M* = 2.57, SD = 1.14) and control condition (*M* = 2.56, SD = 0.82, *p* = 1). There was a marginal significant difference between the flicker-visible condition (*M* = 2.27, SD = 0.82) and the control condition (*p* = 0.054).

Therefore, the flicker-invisible condition (stimulated at 55.5 and 62.5 Hz) showed a moderate effect of time dilation when compared with the control. The flicker-visible condition (stimulated at 6.9 Hz) indicated a strong effect of dilation, which was consistent with previous studies.

#### EEG results

**Figure 10.**
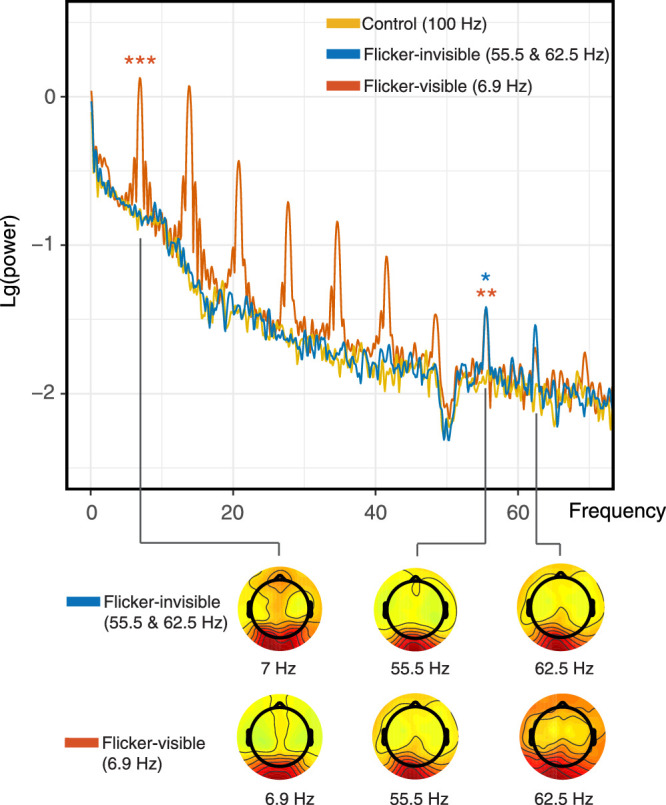
Grand-average log-transformed power spectra for the standard stimulus duration (2 seconds) at Oz. Only the flicker-visible condition showed significant peaks at 6.9 Hz and 55.5 Hz compared with others. Note that we conducted statistical comparisons only at 7 Hz, 55.5 Hz, and 62.5 Hz (*n* = 17). The lower panel shows the power topographies at the frequencies of interest.

After pre-processing, we computed a Fast Fourier Transform (FFT) for individual channels to transform waveforms into the frequency domain. With a sampling rate at 1000 Hz and zero-padding, we computed amplitude spectrums (µV) at values ranging from 0 to 500 Hz in increments of 0.1 Hz. The occipital channel (Oz) had the highest power for all stimulated frequencies (6.9 Hz, 55.5 Hz, and 62.5 Hz) in all conditions; thus, we focused on the Oz channel in the following analysis. The base-10 log-transformed amplitude spectrum was averaged across trials, and then grand-averaged across all participants (Figure 10).

To test whether the flickers induced the corresponding SSVEPs, we conducted separate rmANOVAs on three frequency amplitudes of interest (7 Hz, 55.5 Hz, and 62.5 Hz) with flicker condition as a factor. Note that in the 7 Hz power comparisons in the following analyses, we compared the amplitude at 6.9 Hz in the visible-flicker condition with the amplitude at 7 Hz in the other two conditions because the stimulated frequency in the visible-flicker condition was 6.9 Hz under the 0.1 Hz resolution. For simplicity, we refer to this comparison as between “amplitudes at 7 Hz” in the following text.

At 7 Hz, Mauchly's test of sphericity on the amplitudes from three conditions was significant (*p* = 0.03); thus, we used the Greenhouse-Geisser correction on the following tests. There was a significant effect of flicker condition on amplitudes at 7 Hz (*F*(_1.46, 23.32)_ = 132.78, *p* < 0.001, *Ƞ^2^_p_* = 0.89). Three two-tailed planned comparisons with Bonferroni corrections revealed that the amplitude of 6.9 Hz in the flicker-visible condition (*M* = 0.03, SD = 0.32) was significantly larger than that in the flicker-invisible condition (*M* = -0.88, SD = 0.29, *p* < 0.001, Cohen's d = 2.86) and control conditions (*M* = -0.82, SD = 0.24, *p* < 0.001, Cohen's d = 3.31). However, the amplitude of 7 Hz in the flicker-invisible condition, which represented its beat amplitude, was not significantly larger than that in the control condition (*p* = 0.44). At 55.5 Hz, the test of sphericity was not violated. There was a significant effect of flicker condition on amplitude at 55.5 Hz (*F*_(2, 32)_ = 15.16, *p* < 0.001, *Ƞ^2^_p_* = 0.49). Three two-tailed planned comparisons with Bonferroni corrections revealed that power in the flicker-invisible condition (*M* = -1.60, SD = 0.39) was significantly larger than the control condition (*M* = -2.11, SD = 0.44, *p* = 0.002, Cohen's d = 1.00). The flicker-visible condition (*M* = -1.54, SD = 0.37) was also significantly larger than the control (*p* < 0.001, Cohen's d = 1.27). There was no significant difference between flicker visible and invisible conditions. At 62.5 Hz, there was no significant difference among the three conditions (*F*_(2, 32)_ = 1.43, *p* = 0.25); thus, we conducted no further comparisons.

**Figure 11.**
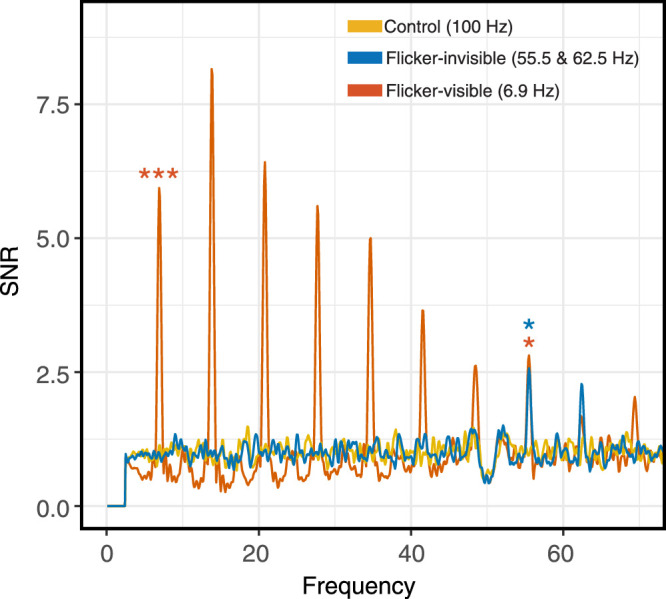
Signal-to-noise spectra of three conditions calculated from power. Note that at 55.5 Hz, the flicker-invisible condition had significantly larger SNR than that of the control. We conducted statistical analyses only at 7 Hz, 55.5 Hz, and 62.5 Hz (*n* = 17). SNR = signal to noise ratio.

We further examined the signal-to-noise ratio (SNR) because it could enhance SSVEP peaks for visualization (Vialatte, Maurice, Dauwels, & Cichocki, 2010). We calculated the SNR by taking the value at each frequency bin divided by the average value of the 20 neighboring bins (Boremanse, Norcia, & Rossion, 2013). As shown in Figure 11, results were similar to the previous analysis. In post hoc two-tailed comparisons between conditions with Bonferroni corrections, the SNR of the flicker-invisible condition (*M* = 2.57, SD = 1.87) was significantly larger than that of the control condition (*M* = 0.95, SD = 0.72, *p* = 0.018, Cohen's d = 0.77). The flicker-visible condition that showed significantly larger power also had larger SNR (*M* = 2.81, SD = 1.78) than that of the control (*p* = 0.002, Cohen's d = 1.03).

Finally, we computed the ITC to examine the direct measurement of phase alignment when neural entrainment occurs. Zoefel et al. (2018) differentiated neural entrainment from repetitive ERPs, suggesting that neural entrainment does not necessarily induce larger power at the stimulated frequency as repetitive ERPs do; rather, it entails phase alignment of endogenous neural oscillation and stimulation. Therefore, ITC analysis that measures the phase coherence provides a better account of neural oscillation strength apart from a power analysis. We, therefore, conducted ITC analysis by taking phase angles from FFT outputs, averaging them across trials following the formula below (Cohen, 2014, p. 244).

$$ITPC_{tf}=\left|n^{-1}\sum_{r=1}^{n}e^{ik_{tfr}}\right|$$

**Figure 12.**
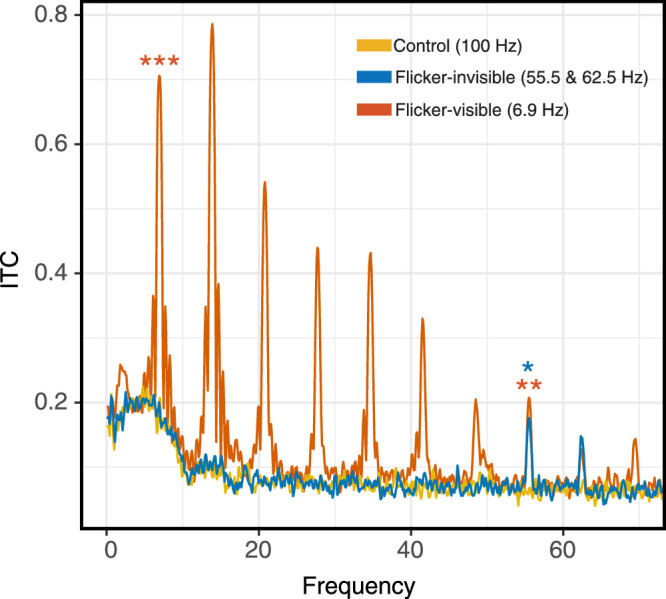
Grand-average ITC spectra of three conditions at Oz. Similar to SNR results, ITC of the flicker-invisible condition was significantly larger than that of the control condition at 55.5 Hz. Again, in the flicker-visible condition, ITC was significantly larger at 7 and 55.5 Hz when compared with other conditions. We conducted statistical analyses only at 7 Hz, 55.5 Hz, and 62.5 Hz (*n* = 17). ITC = inter-trial coherence.

The resulting values were bound between 0 and 1, with 1 referring to the completely identical phase angles across trials, and 0 representing the completely uniform distributed phase angles. We then averaged ITC values across individuals and plotted the ITC spectra by frequency (Figure 12).

An rmANOVA was conducted on three frequencies of interest to compare ITC values across conditions. At 7 Hz, there was a significant effect of flicker condition on ITC values (*F*_(1.20, 19.23)_ = 113.91, *p* < 0.001, *Ƞ^2^_p_* = 0.88). Three two-tailed planned comparisons with Bonferroni corrections found that the 6.9 Hz ITC in the flicker-visible condition (*M* = 0.71, SD = 0.22) was significantly larger than the 7 Hz ITC in the flicker-invisible condition (*M* = 0.19, SD = 0.10, *p* < 0.001) and control condition (*M* = 0.21, SD = 0.09, *p* < 0.001). The 7 Hz ITC in the flicker-invisible condition was not significantly different from that of the control condition. At 55.5 Hz, the test of sphericity was not violated, and there was a significant effect of flicker condition on ITC (*F_(__2, 32)_* = 7.07, *p* = 0.003, *Ƞ^2^_p_* = 0.31). Three two-tailed planned comparisons with Bonferroni corrections revealed that the flicker-invisible condition ITC (*M* = 0.18, SD = 0.15) was significantly larger than the control condition ITC (*M* = 0.07, SD = 0.05, *p* = 0.03, Cohen's d = 0.70). In addition, the flicker-visible condition ITC (*M* = 0.21, SD = 0.13) was significantly larger than the control condition ITC (*p* = 0.003, Cohen's d = 0.97). At 62.5 Hz, there was no main effect of condition on ITC (*F*_(2, 32)_ = 2.43, *p* = 0.10); thus, no further comparisons were conducted. The ITC results were consistent with the power analysis.

### Discussion

In the behavioral data, we observed a moderate extent of dilation in the flicker-invisible condition, suggesting that combinative high-frequency flickers could also induce moderate time dilation. There was a significant extent of dilation in the flicker-visible condition, confirming results of the previous experiments and indicating that saliency was a strong factor in flicker-induced perceived dilation.

In the EEG data, we did not observe a spectral peak at the beat in the flicker-invisible condition. However, further analysis revealed a significantly larger power, SNR and ITC at 55.5 Hz in the flicker-invisible condition compared with that of the control. It is conceivable that, although there was some neural entrainment, the SNR was small especially for high frequencies and the beat, and, therefore, was not adequately captured in our current measurements.

## Experiment 5

Experiment 4 found that combinative high-frequency flickers, or invisible flickers, induced time dilation, although there was no clear observation of the entrainment at the beat. We further conducted an independent psychophysical experiment to test the alternative possibility that participants might perceive invisible flickers as not stable. In this task, we examined whether participants could distinguish between an invisible flicker (55.5 Hz and 62.5 Hz) and stable light (125 Hz). We hypothesized that participants could not distinguish invisible flickers from stable light, so that their accuracy would be lower than or around the chance level. Because there were only two options, the chance rate would be 0.5.

### Participants

Ten new participants were recruited for the discrimination task. They were students from Tokyo University and did not participate in the previous experiments (6 men and 4 women, mean age = 23.3 years, SD = 2.75).

### Methods

**Figure 13.**
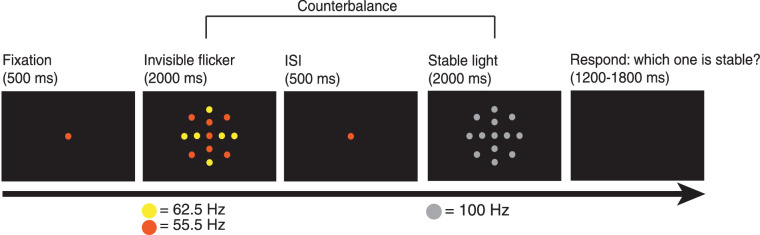
A typical trial in the stableness comparison task. We counterbalanced the order of the invisible flicker and stable flicker across trials. ISI = inter-stimulus interval.

In the stableness discrimination task (Figure 13), participants were sequentially presented with an invisible flicker and a stable stimulus of the same duration. We counterbalanced the order of stimulus across 50 trials. Participants fixated on the center dot and identified which stimulus was more stable by pressing the corresponding keys.

### Results

**Figure 14.**
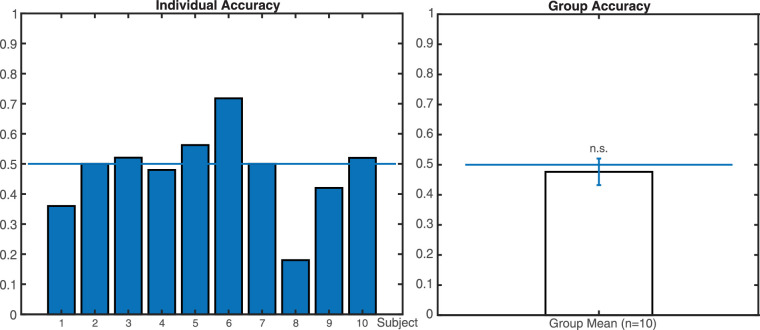
The individual and group mean accuracy for the stableness discrimination task. The error bar represents standard errors of the mean. The group mean did not significantly differ from 0.5, the chance rate (*n* = 10, *p* = 0.301).

As shown in Figure 14, the individual accuracy was the proportion of correct trials out of all non-missing trials. Because participants had only two options in this task, the chance rate was 0.5. We conducted a one-tailed one-sample *t*-test, testing against the alternative hypothesis that the accuracy mean was lower than 0.5. The results did not reject the null hypothesis, suggesting that there was no significant difference in accuracy between participants’ performance (*M* = 0.48, SD = 0.14) and the chance rate (0.5; *t*_(9)_ = -0.541, *p* = 0.301). We further conducted a Bayesian one-sample *t*-test against the alternative hypothesis that the accuracy was larger than 0.5. BF_10_ was 0.22, suggesting moderate evidence for the null hypothesis that the mean accuracy was the same as the chance rate of 0.5.

### Discussion

Participants responded to this discrimination task between invisible flicker and stable light at the chance rate, suggesting that participants could not discriminate between the invisible flicker and stable light and perceived both as stable. This result provided an objective measure of invisibility for the invisible flickers and suggested it was unlikely that the perception of flickering caused the dilation effect observed in Experiment 4.

## General discussion

We conducted separate examinations of the saliency and neural entrainment accounts of time dilation to elucidate the degree to which these two factors determine the effects of flicker-induced time dilation. The first two experiments revealed that the extent of saliency correlated with time dilation, supporting the critical role of saliency in perceiving subjective duration as revealed in previous studies (Herbst et al., 2013). The last three experiments took advantage of the beat frequency to rule out saliency effects and found an effect of combinative high-frequency flickers on time dilation, suggesting that neural entrainment without conscious perception may also influence time perception.

### Saliency determines the perceived duration

Our first two experiments on saliency directly supported the saliency account; change of saliency determined perceived duration. This account rests on two assumptions. First, only stimuli with salient changes can induce perceived dilation, whereas stimuli that lack noticeable changes cannot produce time dilation. Second, durations would be overestimated most at frequencies with the most salient changes. A previous study by Herbst et al. (2013), supported the first inference but not the second, whereas our data validated both assumptions and further highlighted the role of saliency in time perception. Specifically, Herbst et al. (2013) reported a linear decrease in duration overestimation as the frequency increased from 3 Hz; when the frequency was higher than the CFF (stimulation frequency = 55.4 Hz, mean CFF = 50 Hz), the dilation effect disappeared even though SSVEPs still existed in this range. Their results showed that saliency, and not SSVEP, was necessary for perceiving dilation. Regarding the correlation between saliency and perceived dilation, the saliency account hypothesized the strongest dilation at the highest salient frequencies (8 to 15 Hz). Thus, it predicted an inverse U-shape perceived dilation as frequency increases, peaking around 8 to 15 Hz. However, Herbst et al. (2013) did not observe this correlation. Our first two experiments filled this gap by replicating the critical effects of saliency on perceived dilation and further demonstrated that the subjective saliency strength correlated with the extent of perceived dilation. Note that we manipulated the subjective saliency strength with the number of salient flickers, instead of assuming the subjective saliency of flickers at specific frequencies. The results of our first two experiments supported the saliency account, revealing that subjective saliency predicts perceived dilation and the strength of overestimation.

Saliency is critical in time perception, possibly because it increases the level of attention in the interval timing system. The information-processing model of interval timing considers the internal clock as a triad, including the clock, memory, and decision stages (Allman, Teki, Griffiths, & Meck, 2014; Church, 1984; Gibbon, Church, & Meck, 1984; Treisman, 1963). In the clock stage, when the internal clock registers the duration of an external stimulus, pulses are produced by a pacemaker, transmitted through a switch governed by the attention, and counted by the accumulator. This pacemaker - switch - accumulator triad is considered the internal clock that represents stimulus duration. In the memory stage, the current pulse in the working memory is compared with the previous values in the reference memory. Finally, in the decision stage, a judgment is made, and feedback is generated. Within this framework, saliency may influence the clock stage by increasing the level of attention, thus causing pulses to accumulate faster. Indeed, the interference of saliency through the attentional switch in the clock stage has been widely applied to explain temporal distortions. For example, auditory intervals are usually perceived as longer than visual intervals despite the same physical duration (Lustig & Meck, 2011). Such auditory-visual differences are often attributed to the larger salience of auditory stimuli, which distorts the attentional switch and causes a faster accumulation of pulses, thus producing an overestimation of auditory intervals (Allman et al., 2014; Merchant et al., 2013; Repp & Penel, 2002).

### Neural entrainment had only moderate effects

In the second set of experiments, the 71.4 Hz and 83.3 Hz pair yielded no effect on perceived dilation, and the 55.5 Hz and 62.5 Hz pair produced a moderate effect. Only the latter suggests that neural entrainment may affect time perception. The difference in results could only be attributed to the two changes made to the paradigm: the lower frequencies and more integrated stimuli in Experiment 4. Only the lower frequency pair used in Experiment 4 caused time dilation, demonstrating that neural entrainment strength decreases as frequencies increase (Herrmann, 2001; Pastor et al., 2003). Moreover, the absence of time dilation in Experiment 3 may also result from the possibility that the stimuli were not perceived as integrated or coherent enough to arouse the beat. Boremanse et al. (2014) reported that the beat was absent when the two halves of a face stimulus were misaligned or separated. Alp et al. (2018) also revealed that conditions without global integration (i.e. scrambled control and rotated control) had smaller beats compared with those using reflection symmetry stimuli. It may be necessary to ensure the subjectively coherent perception of the stimuli when using visual stimuli to induce the beat.

One feasible cause of the time dilation in the flicker-invisible condition of Experiment 4 is the neural entrainment of the flicker frequencies. First, the stimulus design of combinative high-frequency flickers, or invisible flickers, precluded the effect of change saliency on the dilation. The two fundamental frequencies (55.5 Hz and 62.5 Hz) of invisible flickers were higher than the CFF measured in Experiments 1 and 2, as well as those recorded in the literature (generally 30 Hz to 40 Hz; Eisen-Enosh, Farah, Burgansky-Eliash, Polat, & Mandel, 2017). Thus, these two fundamental frequencies per se were unlikely to be perceived as flickering and cause duration overestimation. Second, Experiment 5 substantiated that participants were likely to perceive invisible flickers stable, as they were unable to distinguish between invisible flickers and stable stimulus. These results suggested that invisible flickers were unlikely to increase saliency. Therefore, it was conceivable that the entrainment of the beat at 7 Hz (f2–f1: and 62.5–55.5 Hz) could have induced such dilation effects. The entrainment of fundamental frequencies indicated by power and SNR also supported this account. Another possibility is that participants might have perceived flickers in some trials that produced weak dilation. However, participants in Experiment 4 did not verbally report perceiving weak flickers, nor did participants in Experiment 5 systematically perceive flickering of the same stimulus. Therefore, this explanation is less probable than the entrainment account.

The neural entrainment account is consistent with the SBF model, which is composed of cortical oscillators at different frequencies and coincidence detectors (Matell & Meck, 2004; Meck, Penney, & Pouthas, 2008; Van Rijn, Gu, & Meck, 2014b). It is presumed that cortical oscillators have an intrinsic frequency that determines their firing probability and that they converge onto spiny neurons that detect the coincidence of oscillatory patterns, which generates an internal representation of duration. Hashimoto and Yotsumoto (2015) incorporated the neural entrainment of external stimuli into the SBF model, which successfully predicted the correspondence between perceived duration and characteristics of the stimuli frequency spectrum. In sum, flickers may induce dilation by entraining the intrinsic oscillators to an external frequency, which consequently modulates neuronal firing patterns and advances the activation of coincidence detector neurons. Our behavioral results were in line with these theories, supporting the account that neural entrainment may influence time perception. Admittedly, the entrainment effect on time dilation observed in Experiment 4 was moderate, which may have resulted from weak entrainment of high fundamental frequencies and the beat. This result was consistent with the Hashimoto and Yotsumoto (2018) finding that a smaller amplitude of the ERP component stimulated frequency corresponded to a shorter reproduced dilation.

The behavioral results differed from the predictions of the Herbst et al. (2013) saliency account. The main discrepancy between the saliency account and the neural entrainment account is the effect of flicker change visibility on perceived duration. In the case of combinative high-frequency flickers whose changes were invisible, the saliency account would predict no dilation. In contrast, the neural entrainment account would predict dilation depending on the strength of neural entrainment. Experiment 4 and Experiment 5 together found that such combinative high-frequency flickers were perceived as stable, and they induced time dilation. Because we controlled the saliency level between the flicker-invisible condition and the control condition, saliency was unlikely to be the factor leading to this result. The result could be better explained by the neural entrainment account.

In the EEG power analyses, the power was not larger at 7 Hz in the flicker-invisible condition than the control condition. This result was inconsistent with our hypothesis and behavioral data, which proposed that the flicker-invisible condition should have stronger power at the beat frequency to induce dilation. This finding may have resulted from the absence of sensory inputs at the beat frequency (Zoefel, ten Oever, et al., 2018). It has been suggested that in neural entrainment, power signals tend to decrease after FFT because phase-resetting of the ongoing oscillations violates the FFT's stationarity assumption. This decrease is usually compensated for by an increase in power from evoked neural activities. Thus, endogenous oscillations without such sensory-evoked activities may be reflected as a decrease in power in the time-frequency analysis. Moreover, the flicker-invisible condition showed a significantly larger SNR and ITC compared with the control only at 55.5 Hz but not 62.5 Hz, despite the larger grand-average means for both frequencies, as shown in the spectrum. Such results indicated weak entrainment of high frequencies. As mentioned above, the neural entrainment of exogenous stimuli weakens as the stimulated frequency increases to gamma band (Gulbinaite et al., 2019). Most studies that observed the beat frequency used low fundamental frequencies, and the resulting IM components, including the beat, still had a smaller amplitude or SNR compared with those of fundamental frequencies (Alp, Kogo, Van Belle, Wagemans, & Rossion, 2016; Boremanse, Norcia, & Rossion, 2013; Boremanse et al., 2014; Sutoyo & Srinivasan, 2009). Our study was designed to test fundamental frequencies above the CFF to preclude saliency, thus rendering neural entrainment even harder. In addition, the beat (sometimes termed the difference IM component) is hard to measure compared with the sum IM components. For example, Cunningham et al. (2017) used two gratings at 2.3 Hz and 3.75 Hz and measured the IM component amplitudes in conditions when gratings formed coherent plaids and non-coherent conditions. The difference component (f2–f1: 1.45 Hz) was not conspicuous above the noise, and there were no differences between the conditions, despite the observation of reliable responses of the sum IM component (2f1 + 2f2) only in the coherent condition. Such observations of lower amplitude or absence of the beat were similar in other intermodulation studies (Aissani, Cottereau, Dumas, Paradis, & Lorenceau, 2011; Alp et al., 2016).

The ITC analysis results were consistent with the power analysis results. We did not observe a larger ITC at the beat frequency in the flicker-invisible condition. This may have been because of weak entrainment or environmental noise and does not necessarily mean that the peak at the beat frequency did not exist. Future research should explore other methods, such as transcranial magnetic stimulation (Thut et al., 2011) to induce stronger neural entrainment to examine the existence of the beat of high frequencies. For example, temporal inference electrical fields have been reported to noninvasively stimulate the deep brain by applying two high frequencies above those of standard neuronal processing and utilizing their difference frequency (Grossman et al., 2017). New noninvasive stimulation techniques provide more possibilities to explore neural entrainment and time perception.

The current study has some limitations. First, in Experiments 3 and 4, although flickers should theoretically stimulate neural oscillations at the beat frequency, our results revealed that such entrainment of the beat was weak and hard to observe. Techniques that ensure strong neural entrainment may provide more conclusive evidence regarding the neural entrainment account. Second, although the standard stimulus duration in the first three experiments was sub-second, Experiment 4 used supra-second durations to obtain better SSVEP observations. Because different brain networks have been reported to engage in sub-second and supra-second interval processing, it is difficult to directly compare the experiments (Hayashi, Kantele, Walsh, Carlson, & Kanai, 2014).

### Conclusions

We observed that subjective saliency is the most critical factor in determining whether time dilation is perceived and the strength of the dilation. Further experiments are needed to examine how attention mediates saliency and time perception, especially through influence on internal clock speed. We also observed a moderate effect of combinative high-frequency flickers on perceived time dilation, suggesting neural entrainment engagement. In other words, flicker-induced time dilation can occur without conscious perception of stimulus changes. Future research should also explore other methods to induce stronger neural entrainment to examine the existence of the beat of high frequencies. In conclusion, the perception of time relies more on later stages of visual processing and psychological aspects and may interact with higher cognitive functions.

## Supplementary Material

Supplement 1
