# Supplementary material for: Effect of change saliency and neural entrainment on flicker-induced time dilation
Source: J Vis. 2020 Jun 23;20(6):15. doi: 10.1167/jov.20.6.15 (PMC7416891; doi:10.1167/jov.20.6.15)
Supplement: Supplement 1 [file jovi-20-6-15_s001.pdf]

Supplementary Materials
Individual PSE parameters and goodness-of-fit for each condition in 4 experiments

Table S1. Psychometric function parameters (threshold, slope, goodness-of-fit) for each individual and each condition in Experiment 1.

The threshold was standardized PSE (i.e., PSE divided by standard duration). Psychometric function for each individual could be constructed with function `PAL_CumulativeNormal` with two fixed parameters (guess rate = 0, lapse rate = 0). We used `PAL_PFML_GoodnessOfFit` to determine the goodness-of-fit, which is the proportion of deviance values from the bootstrap simulations that were greater than the deviance value of the data. Larger values indicate a better fit.

	Flicker-invisible			Combined			Flicker-visible		
	threshold	slope	fit	threshold	slope	fit	threshold	slope	fit
sub01	1.203	0.004	0.929	1.198	0.005	0.244	1.052	0.005	0.333
sub02	1.042	0.006	0.461	1.061	0.011	0.004	0.977	0.006	0.948
sub03	1.141	0.003	0.081	1.155	0.003	0.896	1.051	0.004	0.520
sub04	1.143	0.005	0.177	1.251	0.007	0.475	1.028	0.008	0.321
sub05	1.038	0.008	0.790	1.089	0.006	0.934	1.040	0.008	0.388
sub06	1.083	0.008	0.588	1.043	0.006	0.164	1.029	0.007	0.244
sub07	1.108	0.008	0.598	1.078	0.006	0.228	0.960	0.006	0.956
sub08	1.044	0.006	0.431	1.056	0.005	0.923	0.975	0.005	0.301
sub09	1.101	0.005	0.222	1.201	0.005	0.299	0.993	0.005	0.372
sub10	1.150	0.004	0.440	1.189	0.005	0.848	0.988	0.004	0.969
sub11	0.913	0.007	0.588	0.923	0.007	0.003	0.995	0.007	0.569
sub12	1.012	0.003	0.174	1.146	0.004	0.575	1.025	0.004	0.596

Table S2. Psychometric function parameters (threshold, slope, goodness-of-fit) for each individual and each condition in Experiment 2.

The threshold was standardized PSE (i.e., PSE divided by standard duration). With two fixed parameters (guess rate = 0, lapse rate = 0.02), psychometric function for each individual could be constructed with function `PAL_CumulativeNormal`. We used `PAL_PFML_GoodnessOfFit` to determine the goodness-of-fit, which is the proportion of deviance values from the bootstrap simulations that were greater than the deviance value of data. Larger values indicate a better fit.

	Stable			Combined			Flicker-visible		
	threshold	slope	fit	threshold	slope	fit	threshold	slope	fit
sub01	1.011	0.006	0.628	1.202	0.007	0.911	1.170	0.007	0.740
sub02	0.999	0.008	0.007	1.282	0.006	0.169	1.308	0.005	0.053
sub03	1.050	0.004	0.276	1.114	0.005	0.872	1.119	0.005	0.230
sub04	0.961	0.009	0.316	1.097	0.007	0.059	1.091	0.005	0.240
sub05	0.975	0.008	0.784	1.087	0.007	0.741	1.151	0.006	0.502
sub06	1.024	0.005	0.255	1.077	0.005	0.291	1.138	0.004	0.054
sub07	0.982	0.003	0.526	1.144	0.003	0.132	1.099	0.003	0.759
sub08	1.013	0.006	0.381	1.093	0.006	0.016	1.070	0.007	0.878
sub09	1.006	0.008	0.874	1.031	0.005	0.241	1.048	0.005	0.918
sub10	0.995	0.006	0.329	1.069	0.007	0.019	1.099	0.006	0.266
sub11	0.932	0.007	0.656	0.973	0.007	0.244	0.967	0.006	0.555
sub12	1.051	0.005	0.795	1.116	0.004	0.151	1.136	0.005	0.062

.

Table S3. Psychometric function parameters (threshold, slope, goodness-of-fit) for each individual and each condition in experiment 3.

The threshold was standardized PSE (i.e., PSE divided by standard duration). With two fixed parameters (guess rate = 0, lapse rate = 0), psychometric function for each individual could be constructed with function `PAL_CumulativeNormal`. We used `PAL_PFML_GoodnessOfFit` to determine the goodness-of-fit, which is the proportion of deviance values from the bootstrap simulations that were greater than the deviance value of the data. Larger values indicate a better fit.

	Flicker-invisible			Stable		
	threshold	slope	fit	threshold	slope	fit
sub01	1.039	0.01	0.496	0.998	0.01	0.1
sub02	0.986	0.008	0.077	0.97	0.007	0.459
sub03	1.02	0.004	0.925	0.983	0.004	0.967
sub04	1.013	0.005	0.058	1.105	0.005	0.378
sub05	1.052	0.004	0.424	1.1	0.004	0.052
sub06	0.985	0.004	0.427	1.032	0.005	0.334
sub07	1.021	0.005	0.935	1.039	0.009	0.307
sub08	0.969	0.003	0.258	1.041	0.003	0.617
sub09	1.019	0.005	0.767	0.997	0.006	0.866
sub10	1.019	0.007	0.772	1.001	0.007	0.257

Table S4. Psychometric function parameters (threshold, slope, goodness-of-fit) for each individual and each condition in Experiment 4.

The threshold was standardized PSE (i.e., PSE divided by standard duration). With two fixed parameters (guess rate = 0, lapse rate = 0.02), psychometric function for each individual could be constructed with function `PAL_Logistic`. We used `PAL_PFML_GoodnessOfFit` to determine the goodness-of-fit, which is the proportion of deviance values from the bootstrap simulations that were greater than the deviance value of the data. Larger values indicate a better fit.

	Flicker-invisible			Flicker-visible			Stable		
	threshold	slope	fit	threshold	slope	fit	threshold	slope	fit
sub01	1.065	2.050	0.464	1.113	1.913	0.218	1.030	2.507	0.348
sub02	0.999	5.496	0.580	1.242	4.270	0.394	1.019	4.997	0.478
sub03	0.987	2.978	0.664	1.376	2.151	0.050	1.022	2.013	0.176
sub04	1.076	1.027	0.328	1.150	0.903	0.018	1.004	0.939	0.902
sub05	1.121	1.559	0.780	1.967	1.244	0.496	0.999	1.615	0.696
sub06	0.982	3.321	0.694	1.494	2.092	0.186	0.963	3.526	0.964
sub07	1.037	1.874	0.552	0.861	1.476	0.622	1.066	2.380	0.516
sub08	1.047	2.843	0.598	1.152	2.629	0.532	1.032	2.521	0.712
sub09	1.071	1.785	0.964	1.174	2.724	0.070	0.977	2.319	0.104
sub10	1.018	2.795	0.864	1.551	2.322	0.798	1.017	2.403	0.524
sub11	1.026	2.295	0.028	1.323	1.892	0.144	0.926	2.027	0.222
sub12	1.021	2.349	0.138	1.171	2.248	1.000	0.993	2.302	0.294
sub13	1.088	1.970	0.196	1.295	2.614	0.592	0.970	2.559	0.054
sub14	1.039	4.492	0.118	1.351	3.667	0.658	0.948	4.370	0.836
sub15	0.984	3.206	0.128	1.248	2.214	0.588	0.999	3.219	0.194
sub16	1.133	1.162	0.122	1.267	1.549	0.190	1.032	1.774	0.240
sub17	0.982	2.505	0.662	1.048	2.441	0.698	0.933	2.290	0.636
